# Identifying the Conditions for Cost-Effective Minimally Invasive Neurosurgery in Spontaneous Supratentorial Intracerebral Hemorrhage

**DOI:** 10.3389/fneur.2022.830614

**Published:** 2022-06-02

**Authors:** Floris H. B. M. Schreuder, Mirre Scholte, Marike J. Ulehake, Lotte Sondag, Maroeska M. Rovers, Ruben Dammers, Catharina J. M. Klijn, Janneke P. C. Grutters

**Affiliations:** ^1^Department of Neurology, Center for Neuroscience, Donders Institute of Brain, Cognition and Behavior, Radboud University Medical Center, Nijmegen, Netherlands; ^2^Department of Operating Rooms, Radboud Institute for Health Sciences, Radboud University Medical Center, Nijmegen, Netherlands; ^3^Department for Health Evidence, Radboud Institute for Health Sciences, Radboud University Medical Center, Nijmegen, Netherlands; ^4^Department of Neurosurgery, Erasmus Medical Center, Erasmus MC Stroke Center, Rotterdam, Netherlands

**Keywords:** intracerebral hemorrhage (ICH), health technology assessment (HTA), cost-effectiveness analysis, neurosurgery, minimally invasive surgery (MIS)

## Abstract

**Background:**

In patients with spontaneous supratentorial intracerebral hemorrhage (ICH), open craniotomy has failed to improve a functional outcome. Innovative minimally invasive neurosurgery (MIS) may improve a health outcome and reduce healthcare costs.

**Aims:**

Before starting phase-III trials, we aim to assess conditions that need to be met to reach the potential cost-effectiveness of MIS compared to usual care in patients with spontaneous supratentorial ICH.

**Methods:**

We used a state-transition model to determine at what effectiveness and cost MIS would become cost-effective compared to usual care in terms of quality-adjusted life-years (QALYs) and direct healthcare costs. Threshold and two-way sensitivity analyses were used to determine the minimal effectiveness and maximal costs of MIS, and the most cost-effective strategy for each combination of cost and effectiveness. Scenario and probabilistic sensitivity analyses addressed model uncertainty.

**Results:**

Given €10,000 of surgical costs, MIS would become cost-effective when at least 0.7–1.3% of patients improve to a modified Rankin Scale (mRS) score of 0–3 compared to usual care. When 11% of patients improve to mRS 0–3, surgical costs may be up to €83,301–€164,382, depending on the population studied. The cost-effectiveness of MIS was mainly determined by its effectiveness. In lower mRS states, MIS needs to be more effective to be cost-effective compared to higher mRS states.

**Conclusion:**

MIS has the potential to be cost-effective in patients with spontaneous supratentorial ICH, even with relatively low effectiveness. These results support phase-III trials to investigate the effectiveness of MIS.

## Introduction

Spontaneous intracerebral hemorrhage (ICH) yearly affects more than 3 million people worldwide (1). ICH has a poor prognosis with 1-month case fatality of 40%, which has not improved in the past decades (2–4). Hematoma volume is one of the most important predictors of an outcome after ICH (5). Therefore, research has been focused on neurosurgical treatment to reduce hematoma volume. Earlier trials comparing neurosurgical open craniotomy to standard care have, however, failed to show improvement in a functional outcome (6, 7). As an alternative, minimally invasive stereotactic aspiration and thrombolytic irrigation of the hematoma were studied in the MISTIE-III trial (8, 9). In 506 patients with a hematoma volume of at least 30 ml, intervention (initiated after a mean of 58 h after the ICH onset) did not seem to be superior to standard medical care (8). In spite of these results, a recent systematic review and meta-analysis of surgical treatments for supratentorial ICH have concluded that surgical treatment for ICH still holds promise, especially with minimally invasive methods and when performed early after the symptom onset (10).

Minimally invasive techniques include stereotactic aspiration and endoscopy-guided approaches. The latter technique uses irrigation-aspiration devices to mobilize the hematoma clot, without the use of thrombolytic agents. Several small case-series of endoscopy-guided minimally invasive surgery have suggested that this technique can safely reduce hematoma volume by 54–94% but lacked a control group or did not report a functional outcome beyond hospital admission (11–13). To properly assess its added value, a phase-III randomized clinical trial is needed to demonstrate the effectiveness of this minimally invasive surgical technique to improve the functional outcome of patients with spontaneous supratentorial ICH, when compared to current usual care.

Given the results of previous trials and the meta-analysis, choosing an optimal trial design and patient population (e.g., distribution of an outcome and age) for such a trial is important as these factors can have a large effect on the (cost-) effectiveness of MIS. One tool that can inform trial design is health economic modeling. Modeling can help to determine the minimal effectiveness and maximum cost from a cost-effectiveness perspective and may determine subgroups in which the intervention is likely to be more or less effective. Before starting a phase-III trial, we aim to assess the conditions that need to be met to reach the potential cost-effectiveness of minimally invasive surgery (MIS) in comparison with usual care in three different populations of patients with spontaneous supratentorial ICH.

## Methods

We followed the modeling good research practices and the Consolidated Health Economic Evaluation Reporting Standards (CHEERS) statement guidelines (**Appendix 1**) (14, 15).

### Model Development

**Figure 1:**
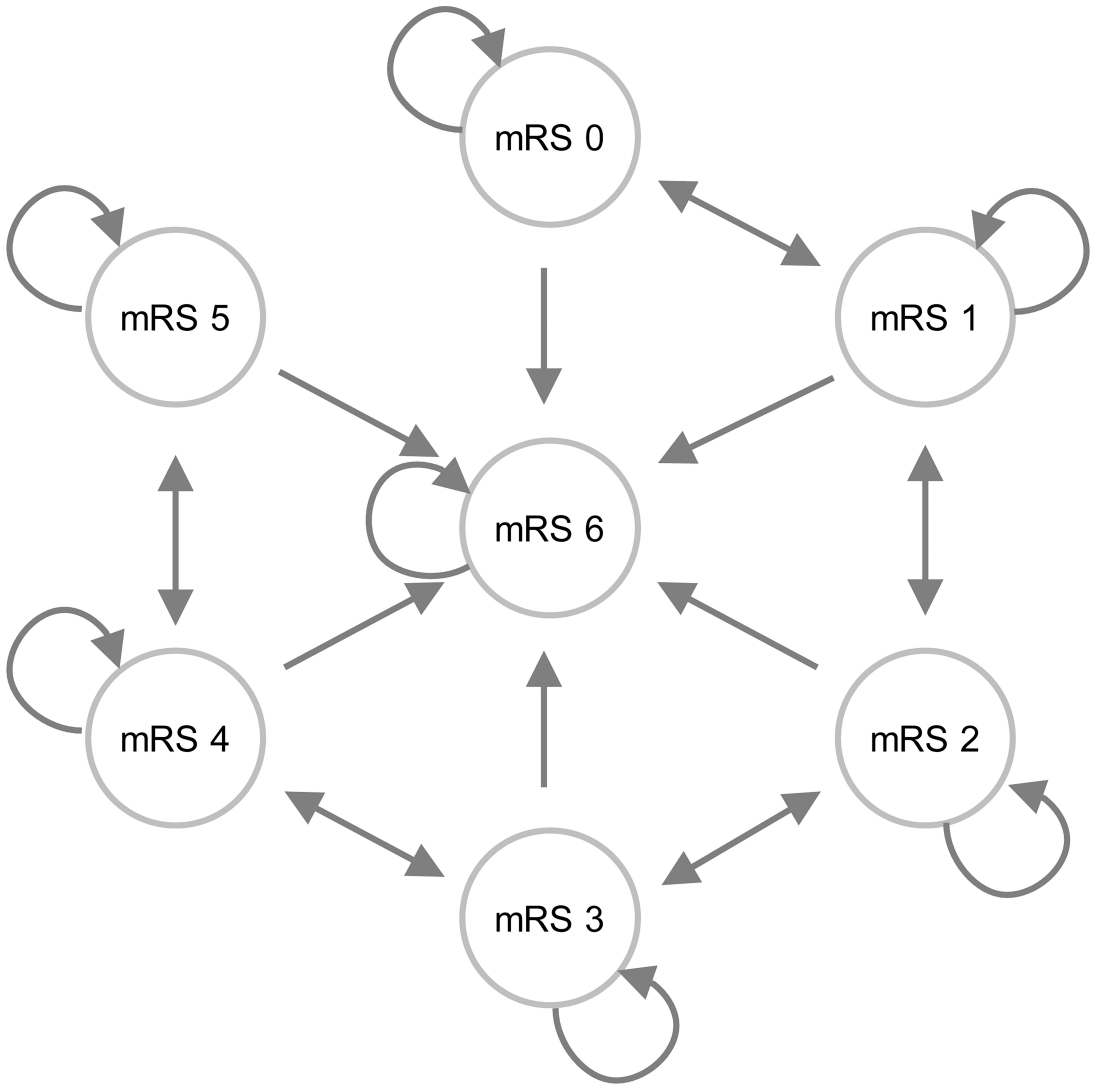
An influence diagram of the state transition model. Patients could enter the model *via* one of the mRS score health states. The arrows indicate the most likely transitions during follow-up; however, it was also possible to improve from mRS 3 to mRS 1, for example.

To evaluate the conditions that need to be met to reach the potential cost-effectiveness of MIS compared to usual care, we constructed a state transition (Markov) model, representing patient follow-up (Figure 1). Health states represented a functional outcome after the index ICH based on the modified Rankin Scale (mRS) score. The mRS is a widely used functional outcome assessment scale, which ranges from 0 (no symptoms) to 6 (death) (16). To simulate the course of the disease and patient follow-up, we built a model with cycles of 3 months. In each cycle, patients had a probability to move to a different health state.

The model reflects the change in health states as a result of the two treatment strategies in 3 hypothetical cohorts of patients with spontaneous supratentorial ICH. The cohorts differ in age, case-fatality, and in the distribution of mRS scores. Cohort 1 was based on a report of the 12-month validation of the ICH score in patients with a mean age of 70 years, in which 33% had an mRS 0–3 (Table 1) (17) Cohort 2 was based on the control group of the Tranexamic acid for the primary intracerebral hemorrhage trial (TICH-2) with an average age of 69 years, in which 46% had an mRS of 0–3 (Table 1) (18). Cohort 3 included the control group of the MISTIE-III trial, which had a mean age of 62 years, in which 11% of the patients had an mRS of 0–3 (Table 1) (8). Because decision-making and an outcome after surgical treatment differ between supratentorial and infratentorial ICH, we restricted our model to supratentorial ICH.

**Table 1:** Initial distribution of patients.

	Cohort 1 *	Cohort 2 **	Cohort 3 ***
	**n (%)**	**n (%)**	**n (%)**
mRS 0	5 (3%)	24 (2%)	0 (0%)
mRS 1	21 (11%)	124 (11%)	0 (0%)
mRS 2	12 (6%)	181 (16%)	9 (4%)
mRS 3	25 (13%)	194 (17%)	16 (7%)
mRS 4	32 (17%)	221 (19%)	60 (25%)
mRS 5	6 (3%)	162 (14%)	124 (51%)
mRS 6	87 (46%)	249 (22%)	34 (14%)

* *Based on 3-month post-ICH results of Hemphill et al. (17);*

** *Based on 3-month post-ICH results of the TICH-2 control group (18);*

*** *Based on 1-month post-ICH results of the MISTIE-III control group (8).*

*ICH, intracerebral hemorrhage; mRS, modified Rankin Scale*.

The usual care strategy was based on the 2015 American Heart Association/American Stroke Association guideline for the management of ICH and consisted of admission to a stroke unit and pharmacological reduction of blood pressure if indicated (19, 20). As open craniotomy is currently performed in only a small minority of patients, this was not included.

We assumed that only index ICH and age affect a functional outcome and transitions between health states. Because we did not expect MIS to affect the number of ICH recurrences or other cardiovascular diseases beyond the 30-day postoperative period (e.g., ischemic stroke, myocardial infarction), recurrent ICH events were not included in our model.

### Transition Probabilities

Transition probabilities determined how the patients could move through different health states in the model. The transition probabilities at 6, 9, and 12 months were derived from the raw data of the 188 conservatively treated patients with supratentorial ICH of Cohort 1 (Table 2) (17). Beyond the 1st year after the index ICH, we assumed that the mRS score was stable for the remaining lifetime. From then on, the patients either stayed in their health state, or died. The probability of dying was based on the mortality rate of the general Dutch population (21), multiplied by the additional risk of dying caused by the ICH. The hazard ratio of dying from ICH-related causes was 0 in mRS 0 and 1, and increased from 1.11 to 2.38 in mRS 2 to 5, respectively (22).

**Table 2:** Transition probabilities.

		Transition probabilities at 6 months post-ICH*						
		**mRS 0**	**mRS 1**	**mRS 2**	**mRS 3**	**mRS 4**	**mRS 5**	**mRS 6**
Health state at 3 months post-ICH	mRS 0	0.80 (*n =* 4)	0.20 (*n =* 1)	-	-	-	-	-
	mRS 1	-	0.95 (*n =* 20)	-	-	0.05 (*n =* 1)	-	-
	mRS 2	-	0.17 (*n =* 2)	0.75 (*n =* 9)	-	-	-	0.08 (*n =* 1)
	mRS 3	-	0.04 (*n =* 1)	0.20 (*n =* 5)	0.68 (*n =* 17)	0.04 (*n =* 1)	-	0.04 (*n =* 1)
	mRS 4	-	-	-	0.16 (*n =* 5)	0.81 (*n =* 26)	0.03 (*n =* 1)	-
	mRS 5	-	-	-	-	-	1.00 (*n =* 6)	-
	mRS 6	-	-	-	-	-	-	1.00 (*n =* 87)
		Transition probabilities at 9 and 12 months post-ICH*						
		**mRS 0**	**mRS 1**	**mRS 2**	**mRS 3**	**mRS 4**	**mRS 5**	**mRS 6**
Health state at 6 or 9 months post-ICH	mRS 0	1.00 (*n =* 4)	-	-	-	-	-	-
	mRS 1	-	0.96 (*n =* 23)	-	-	-	-	0.04 (*n =* 1)
	mRS 2	0.07 (*n =* 1)	0.21 (*n =* 3)	0.64 (*n =* 9)	0.07 (*n =* 1)	-	-	-
	mRS 3	-	-	0.09 (*n =* 2)	0.91 (*n =* 20)	-	-	-
	mRS 4	-	-	-	0.07 (*n =* 2)	0.71 (*n =* 20)	0.18 (*n =* 5)	0.04 (*n =* 1)
	mRS 5	-	-	-	-	-	1.00 (*n =* 7)	-
	mRS 6	-	-	-	-	-	-	1.00 (*n =* 89)
		**Transition probabilities after 1 year post-ICH**						
		**mRS 0**	**mRS 1**	**mRS 2**	**mRS 3**	**mRS 4**	**mRS 5**	**mRS 6**
		We assumed that patients would remain in the health state they were in at 1 year post-ICH and only had a chance to die of ICH related causes [Samsa et al. (21)] or natural causes [Statistics Netherlands (20)].						

* *Based on Hemphill et al. (17).*

*ICH, Intracerebral hemorrhage; mRS, modified Rankin Scale; SD, standard deviation*.

### Effectiveness

Effectiveness was measured in terms of quality-adjusted life years (QALYs). QALYs were constructed by multiplying the utility weight for each health state (an mRS score) with the remaining life years (Table 3). The utility is a score for health-related quality of life and ranges from 0 (death) to 1 (perfect state of health). We derived utility weights per mRS score from a large individual patient data meta-analysis of 4,479 patients with ICH (23). The average utility weight for an mRS score of 5 is negative, which indicates that this health state is considered to be worse than death. QALYs were discounted with an annual rate of 1.5%, according to the Dutch guidelines for economic evaluation (24).

**Table 3:** Utilities.

	Utility score (95% CI)*
mRS 0	0.971 (0.935–1.00)
mRS 1	0.875 (0.862–0.888)
mRS 2	0.742 (0.709–0.775)
mRS 3	0.553 (0.521–0.586)
mRS 4	0.199 (0.167–0.231)
mRS 5	−0.186 (-0.227–0.146)
mRS 6	0

* *Raw data obtained from correspondence to authors of paper by Wang et al. (22).*

*mRS, modified Rankin Scale*.

### Costs

Costs (in Euros) were calculated from the Dutch health care perspective and were adjusted to the price index of 2019 (23). We included direct healthcare costs in our model, i.e., costs for hospitalization, rehabilitation, nursing home care, visits to general practitioner and outpatient departments, medication, home care, and costs that directly result from MIS (Supplementary Table 1) (20) Resource use of usual care was based on a retrospective cohort of consecutive patients (N = 173) with spontaneous supratentorial ICH treated at our hospital between January 2012 and December 2015, supplemented with data from the literature (Supplementary Tables 2–4) (25, 26) The retrospective cohort provided length of stay at the different hospital wards (i.e., intensive care, medium care, stroke unit, or general ward), length of stay at rehabilitation or nursing home facilities, and the number of outpatient visits. Medication costs and resource use for home care up to the 1st year after the index ICH were derived from the literature (25, 26) and were assumed to remain stable over time thereafter. The frequency of visits to the general practitioner was based on the Dutch multidisciplinary guideline for cardiovascular risk management (27). We calculated the average costs using the mean lengths of stay and standard Dutch unit costs (Table 4) (24). Since costs for MIS are not yet known, we set the total surgical costs (including use of operating facilities, surgical staff, device use, surgical supplies, and postoperative care) at €10,000 by default based on an expert opinion. Costs were discounted with an annual rate of 4%, according to Dutch guidelines for economic evaluation (24).

**Table 4:** Costs (in Euro).

	**0–3 months post-ICH (mean, 95% CI)**	**Three-monthly costs between 3 and 12 months post-ICH (mean, 95% CI)**	**Three-monthly costs after 1 year post-ICH (mean, 95% CI)**
mRS 0	16,012 (8,536–26,707)	1,569 (546–3,355)	197 (162–236)
mRS 1	13,026 (7,497–21,375)	3,622 (2,232–5,628)	197 (162–235)
mRS 2	18,447 (12,163–26,970)	6,111 (3,679–9,825)	3,482 (968–7,898)
mRS 3	33,952 (21,742–51,012)	12,476 (7,836–19,587)	181 (132–238)
mRS 4	34,237 (25,196–46,305)	11,136 (7,048–16,371)	1,521 (195–5,029)
mRS 5	72,303 (43,930–113,159)	28,031 (19,521–38,476)	13,269 (8,314–19,285)
mRS 6	5,612 (3,920–7,822)	5.30 (0–26.95)	0 (0–0)

*CI, confidence interval; ICH, Intracerebral hemorrhage; mRS, modified Rankin Scale*.

### Model Validation

We validated the model in accordance with the AdViSHE checklist through consulting clinical experts, cross-validation with relevant literature, checks by independent modeling experts, and extreme value, and subunit testing (28).

### Data Analysis

**Figure 2:**
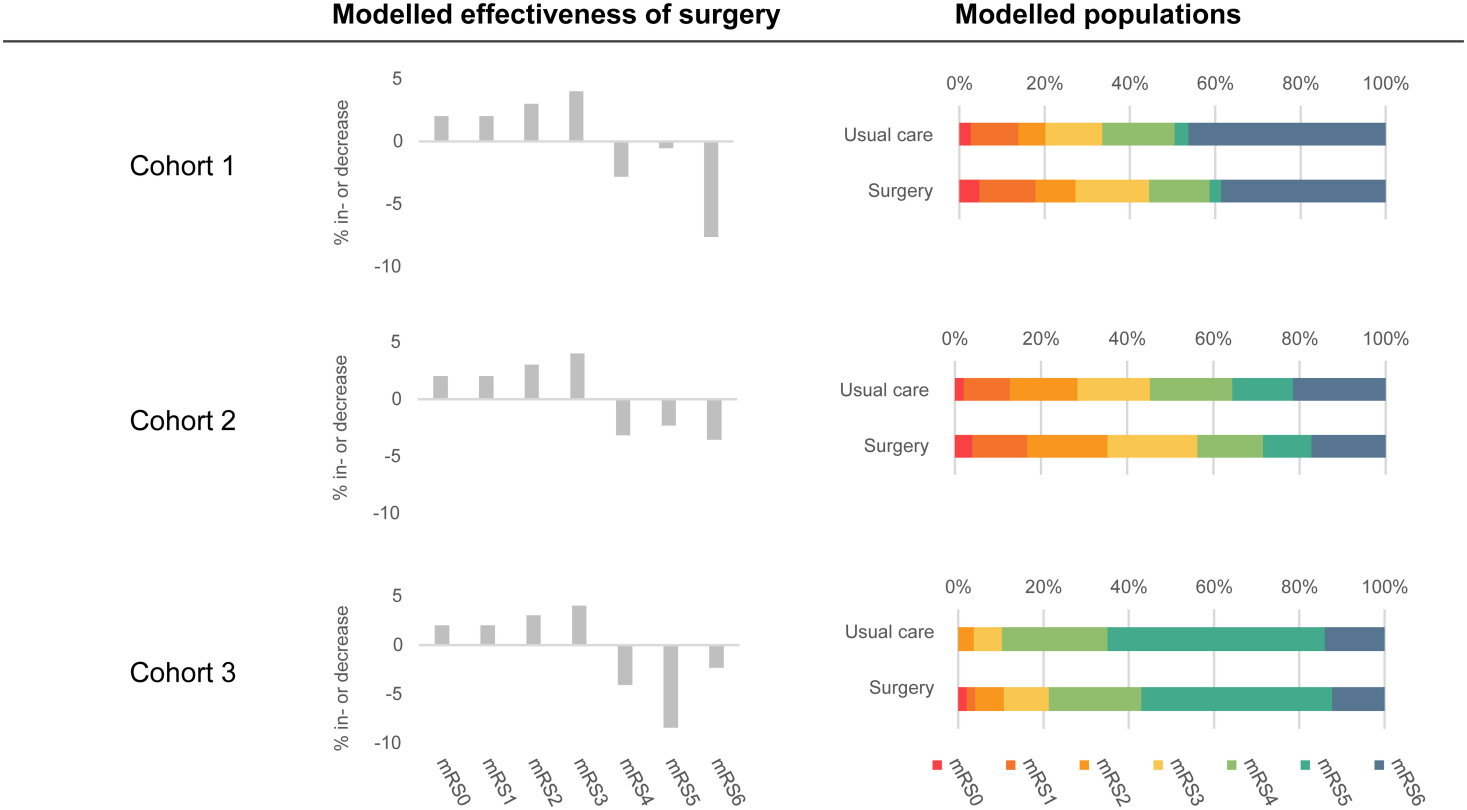
An overview of included populations and modeled effectiveness of MIS. The modeled populations column shows the distributions of the patient populations in both strategies at 3 months post-ICH. The usual care strategy is based on the Hemphill (Cohort 1), TICH-2 (Cohort 2), and MISTIE-III (Cohort 3) populations. The surgery strategy is based on usual care strategy in combination with the modeled effectiveness of surgery (displayed in the middle of the figure). Modeled effectiveness of surgery was based on 11% effectiveness.

To assess the conditions that need to be met to reach the potential cost-effectiveness of MIS in comparison with usual care, we evaluated costs and health outcomes per patient over a lifetime horizon for the three patient cohorts. The effectiveness of MIS was assumed to be an absolute improvement toward mRS 0–3 for the patients treated with MIS compared to usual care, using 11% as an example (Figure 2). We assumed that the patients who improved ended up in mRS 0–3 in an increasing extent (i.e., 18% increase in mRS 0, 18% increase in mRS 1, 27% increase in mRS 2, and 37% increase in mRS 3) and that the decrease in mRS 4–6 was relative to the number of the patients in that state in the original cohort. We can illustrate this best using an example population with an equal percentage of the patients in each mRS state, i.e., 14.3% of the patients per health state. The percentage of the patients in mRS 0–3 is then 57.2% and will increase with 11 to 68.2% due to surgery. The percentage of the patients in mRS 0 increases with 18% of this 11 to 16.3%, mRS 1 increases with 18% of 11 to 16.3%, mRS 2 increases with 27% of 11 to 17.3%, and mRS 3 increases with 37% of 11 to 18.3%. The percentages of the patients in mRS 4, 5, and 6 will all decrease with 1/3 of 11 to 10.6%. The transition probabilities for MIS beyond 3 months post-ICH were considered to be equal to the transition probabilities in the usual care strategy.

We conducted four types of analyses: threshold, two-way sensitivity, scenario, and probabilistic sensitivity analysis. For these analyses, we used the incremental net monetary benefit (iNMB), which was calculated by multiplying the incremental effects (QALYs) of MIS compared to usual care with the willingness-to-pay (WTP) per QALY, subtracting the incremental costs. In the Netherlands, maximum additional costs per QALY range from €20,000 for diseases with a low burden of disease to €80,000 for diseases with a high burden (29). Because of the high disease burden of ICH (1), the highest Dutch cost-effectiveness threshold of €80,000 per QALY was applicable according to the iMTA disease burden calculator (30). An iNMB above 0 means that MIS is cost-effective, whereas an iNMB below 0 implies that it is not. The value of effects or costs where the iNMB is 0 is the threshold for cost-effectiveness. For example, when a new strategy gains 0.1 QALYs extra and costs €1,000 more than the current strategy, the iNMB is 0.1 × 80.000 − 1, 000 = 7, 000. The iNMB is positive, which means that the new strategy is cost-effective. The threshold for cost-effectiveness (iNMB = 0) is found at €8,000 extra costs or 0.0125 gained QALYs for the new strategy compared to the current strategy. All analyses were conducted using R 3.5.3 and the Tidyverse (v1.3.0) package (31).

#### Threshold Analysis

Since both costs and effectiveness of MIS are unknown, we explored at which values of these parameters MIS could potentially be cost-effective. We explored this by threshold analysis, in which we fixed the value of one parameter (e.g., costs) and explored at which value of the other parameter (e.g., effectiveness) MIS becomes cost-effective. By default, we set the surgical costs to €10,000 and the effectiveness to an absolute improvement of 11% in mRS 0–3.

#### Two-Way Sensitivity Analysis

To allow the determination of the cost-effective strategy for each combination of cost and effect, we conducted two-way sensitivity analyses. For this purpose, we ranged the effectiveness of MIS (absolute improvement in the mRS score 0–3) between 0 and 20%, and surgical costs of MIS between €0 and €50,000.

#### Scenario Analysis

In the scenario analysis, we assumed that all the patients receive MIS, but that MIS is effective in a specific health state only, e.g., the patients in mRS 4. It was also assumed that, when the patients improved, they did so by one health state, e.g., mRS 4 patients would transit to mRS 3. The minimal effectiveness of MIS was established by determining at which effect MIS becomes cost-effective, with surgical costs fixed at €10,000.

#### Probabilistic Sensitivity Analysis

Uncertainty with respect to the input parameters was addressed in a probabilistic sensitivity analysis by taking 5,000 samples from the distributions of these parameters. MIS costs and effectiveness were incorporated in the probabilistic sensitivity analysis as fixed values. All results were based on probabilistic outcomes with 95% confidence intervals (95%-CI) calculated using the percentile method.

## Results

In Cohort 1, the usual care strategy gained, on average, 3.5 (95%-CI: 2.8–4.2) QALYs at an average cost of €79,879 (95%-CI: €49,681–€127,262) per patient. Cohort 2 gained, on average, 4.4 (95%-CI: 3.7–5.1) QALYs at an average cost of €153,961 (95%-CI: €90,591–€246,078) per patient in the usual care strategy. Cohort 3 gained, on average, 0.6 (95%-CI: −0.8–1.8) QALYs at an average cost of €433,191 (95%-CI: €212,123–€712,743). The conditions that needed to be met for MIS to become cost-effective were assessed in comparison with usual care.

### Threshold Analysis

For all three populations, we explored the minimal effectiveness and maximum cost at which MIS would be cost-effective. Given €10,000 of surgical costs, MIS became cost-effective when at least 1.3% (95%-CI: 1.2–1.5%) of the patients improved to mRS 0–3 in Cohort 1 (in the usual care strategy, 33.6% had a good outcome, and this increased to 35.1% in the surgical strategy). For Cohort 2, this percentage was 1.1% (95%-CI: 0.9–1.4%), and, for Cohort 3, it was 0.7% (95%-CI: 0.5–0.9%). Given 11% effectiveness of MIS, surgical costs may be up to €83,301 (95%-CI: €71,633–€93,496) for Cohort 1, €164,382 (95%-CI: €120,973–€203,160) for Cohort 2, and €99,581 (95%-CI: €75,749–€118,391) for Cohort 3, for MIS to be cost-effective. Differences between cohorts can be explained by the differences in mRS distributions of poor functional outcomes and the mean age of patients. For example, Cohort 3 had a relatively low threshold, probably due to the high number of the patients in mRS 5 that improve to mRS 0–3 and the relatively young age of this cohort.

### Two-Way Sensitivity Analysis

**Figure 3:**
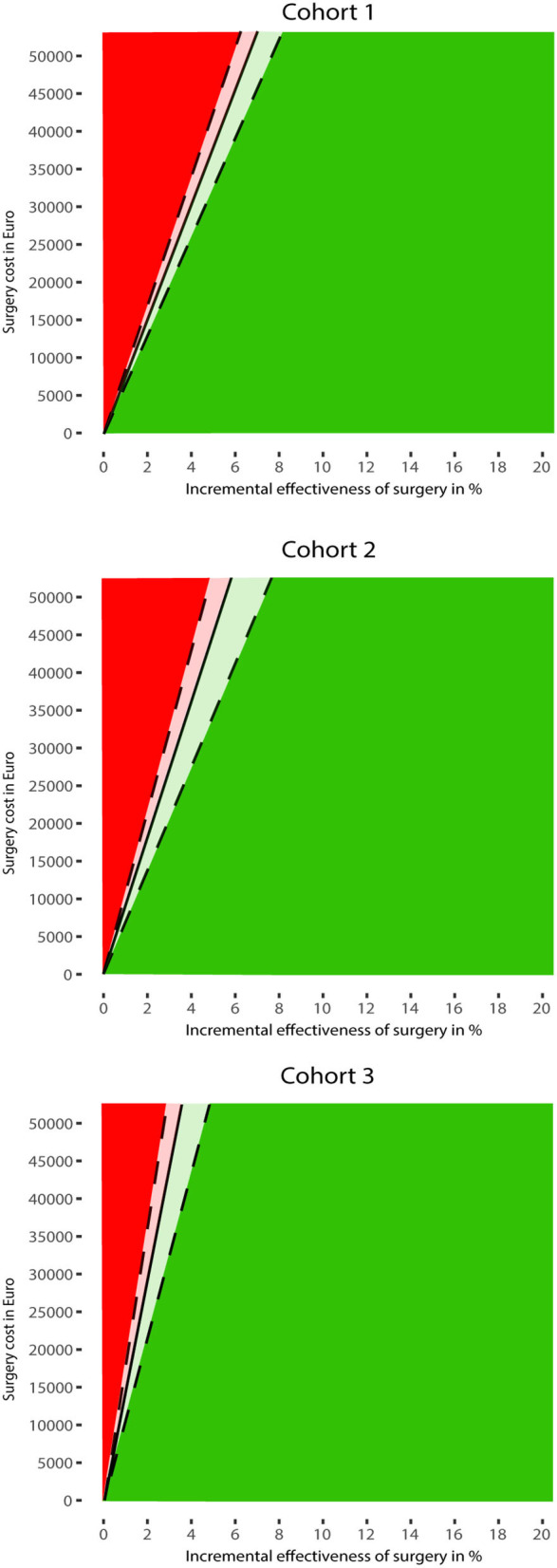
Results of the two-way sensitivity analyses. The MIS strategy is potentially most cost-effective for combinations of surgery cost and effectiveness in the green area. The red area indicates when usual care is most cost-effective. The effectiveness of MIS was assumed to be an absolute improvement toward mRS 0–3 for patients treated with MIS compared to usual care. Both strategies are equally cost-effective at the threshold indicated by the black line. The dashed lines indicate the 95% confidence interval of the threshold line.

Figure 3 shows the two-way sensitivity plot for each cohort. In all cohorts, the cost-effectiveness of MIS was mostly determined by its effectiveness. This indicates that, even when MIS is associated with considerable costs, it would still become cost-effective with low effectiveness. For example, if the effectiveness of MIS is only 1.3%, MIS would be cost-effective with €10,000 treatment costs.

### Scenario Analysis

The minimal effectiveness of MIS to be cost-effective was assessed, when only patients in a particular health state improved by only one mRS state (e.g., only a proportion of mRS 4 patients transit to mRS 3 due to surgery). Given €10,000 of surgical costs, MIS would never become cost-effective when only mRS 6 patients transit to mRS 5. This is because patients in mRS 5 had a worse utility score and higher costs compared to mRS 6 patients. MIS is most cost-effective when mRS 5 patients transit to mRS 4. At least 1.5% (95%-CI: 0.8–2.5%; Cohort 1), 1.5% (95%-CI: 0.8–2.5%; Cohort 2) or 1.1% (95%-CI: 0.6–1.7%; Cohort 3) of patients need to improve from mRS 5 to mRS 4 to be cost-effective compared to current care. Thresholds increase when patients with a lower mRS state were treated, with highest thresholds for patients in mRS1 who transit to mRS 0. Then, at least 5.3% (95%-CI: 2.4–10.%; Cohort 1) or 5.3% (95%-CI: 2.4–10.%; Cohort 2) of patients need to improve from mRS 1 to mRS 0 to be cost-effective compared to current care. It was not possible to obtain a threshold for Cohort 3, as there were zero patients in mRS 1 in this cohort.

## Discussion

In patients with spontaneous supratentorial ICH, minimally invasive surgery has the potential to be cost-effective, even with relatively low effectiveness. Assuming €10,000 of surgical costs, MIS would become cost-effective when at least 0.7–1.3% of the patients improve to mRS 0–3 compared to usual care, depending on the population characteristics. When 11% of the patients improve to a favorable functional outcome, surgical costs may be up to €83,301–€164,382. The cost-effectiveness of MIS was mainly driven by its effectiveness. In lower mRS states, MIS needs to be more effective to be cost-effective compared to higher mRS states.

Cross-validation of our model with relevant literature showed that results of the usual care strategy were comparable. We have established the lifetime average number of QALYs of the patients with supratentorial ICH treated with usual care between 0.6 and 4.4, which is most comparable to the published QALYs of 2.8–4.7 after supportive treatment in patients with ICH (32, 33). The QALYs in Cohort 3 were lower compared to the literature, whereas the lifetime costs for the patients in Cohort 3 were higher, which was caused by a larger proportion of the patients with an mRS score of 5 (Figure 2). Lifetime costs of the usual care strategy ranged between €79,879 and €433,191 in our model, which corresponds to €49,081 to €189,744 (using the average 2019 exchange rate of 0.8931)(34) ^1^, while, in previous studies, these costs ranged from $54,956 to $212,456. (32, 35–37) In general, differences between costs can be explained by different cost perspectives (26, 32, 36, 38), different treatments (38), or different patient populations (26, 35, 37, 39). Our study has a number of strengths. First, we performed our analysis *prior* to the start of a multicenter phase-III randomized clinical trial. Our modeling study provides valuable insights into patient populations that could benefit most from surgery and minimum effectiveness and maximum costs from a cost-effectiveness perspective. This helps in the design of the phase-III randomized clinical trial. Given the neutral results of recent trials, optimal study design and planning are especially important. Additionally, we adhered to guidelines on (model-based) economic evaluation, and combined evidence from the literature, with data from current clinical practice. To determine the influence of the uncertainty of the input parameters, we performed a probabilistic sensitivity analysis.

Our study also has some limitations. First, transition probabilities and costs were based on relatively small cohorts that were divided in seven categories of mRS scores. In our model, the costs for patients with an mRS of 3 between 3 months and 1 year were based on only 8 patients, and costs were lower than expected based on increasing costs per mRS score. These 8 patients had a relatively good recovery and were discharged to home instead of to a rehabilitation center or nursing home, which resulted in lower costs. Nevertheless, the transition probabilities in our data were comparable to a recently published study of 173 patients who were followed up to 12 months after ICH (40).

Second, the three cohorts were slightly different. Cohort 3 had follow-up data available at 1 month, instead of 3 months for Cohorts 1 and 2. Additionally, Cohort 2 also included infratentorial hemorrhages (7% of total) (18), which may have influenced mRS status. Changes in mRS states at 6, 9, and 12 months were only available for Cohort 1; therefore, we used these probabilities for all three cohorts.

Third, we assumed that hospital length of stay was not influenced by therapeutic strategy other than through differences in a functional outcome. This seemed reasonable to assume, since in MISTIE-III time of ICU admission, was similar between surgically treated patients and patients in usual care (8).

Fourth, we found that the largest impact on cost-effectiveness can be achieved in the higher mRS scores, but it remains difficult to predict the eventual outcome at admission. Several prediction models exist to estimate a functional outcome, however with moderate performance (5, 41). Thus, predicting a functional outcome on an individual basis remains rather inaccurate. Based on our results, surgery should refrain from reducing mortality at the cost of an increase of subjects with mRS of 5, as this results in higher costs and lower QALYs.

Lastly, our model included Dutch healthcare costs, which might limit the applicability to generalize our results to other healthcare systems. However, as we provided extensive details of the input parameters in the model, interested readers can assess the transferability of the results to their specific situation (42) In our model, we only included direct medical costs, e.g., costs resulting from hospital admission, rehabilitation, and medical treatment. We decided not to include indirect costs resulting from the ICH, such as loss of productivity from patients and their caregivers. Because we included only direct healthcare costs, we probably have been conservative and underestimated the cost-effectiveness of MIS.

Given the neutral results of the MISTIE-III trial, one might question whether new trials aimed at reducing hematoma volume are needed. Based on the recent meta-analysis, timing of surgery may modify the effect of surgery (10). In the three large, international, multicenter, randomized clinical trials on neurosurgical treatment of ICH (STICH, STICH-II, and MISTIE-III), average time between the symptom onset and surgery ranged from 26 to 58 h (6–8). Earlier surgery might be more beneficial, as hematoma growth occurs in up to a quarter of patients in the 1st h after the ICH onset (10, 43). Hence, further randomized trials are needed to demonstrate whether minimally invasive surgery performed *early* after the symptom onset improves a functional outcome after ICH. Several trials using minimally invasive surgery are ongoing or being scheduled (Table 5), of whom some investigate MIS early after the symptom onset. By estimating the potential value of MIS in this modeling study, we have shown that societal investments in phase-III randomized clinical trials are justified to achieve a high level of evidence for the use of neurosurgical treatment of patients with ICH. Once the results of these trials become available, these can be incorporated into the model to estimate the actual cost-effectiveness of MIS.

**Table 5:** An overview of ongoing or scheduled randomized trials using minimally invasive surgery in intracerebral hemorrhage.

**Study**	**Country**	**Intervention**	**Time window**	**Sample size**	**Primary endpoint**	**Status**
ENRICH NCT02880878	USA	NICO BrainPath® and Myriad®	< 24 h	300	Utility-weighted mRS at 6 months	Start December 2016
MIND NCT03342664	USA, Canada, Germany	Penumbra Artemis® Neuro Evacuation device	< 72 h	500	Ordinal shift analysis of mRS at 6 months	Start February 2018
EVACUATE NCT04434807	Australia	Aurora® Surgiscope	< 8 h	240	Dichotomized mRS 0–3 vs. 4–6 at 6 months	Start November 2020
DIST	Netherlands	Artemis® NeuroEvacuation device	< 8 h	600	Ordinal shift analysis of mRS at 6 months	Start 2022

*DIST, Dutch intracerebral hemorrhage surgery trial; ENRICH, early minimally invasive removal of intracerebral hemorrhage; EVACUATE, ultra-early, minimally invasive intracerebral hemorrhage evacuation vs. standard treatment; MIND, a minimally invasive Neuro evacuation device in the removal of intracerebral hemorrhage; mRS, modified Rankin Scale*.

Our results have several implications for the design of future phase-III clinical trials investigating the potential effectiveness of MIS in patients with ICH. First, the effectiveness of MIS may be as low as 0.7% to become cost-effective, which indicates that, even a very small improvement in a functional outcome could be deemed worthwhile from a societal perspective. However, this should not be mistaken for a clinically meaningful difference with a corresponding number needed to treat. Second, our study provides insight where the largest impact on cost-effectiveness can be achieved, i.e., in higher mRS states (mRS 4 and 5). Our results illustrate that a transition from mRS 6 to mRS 5 will have a negative impact on cost-effectiveness. As cost-effectiveness of MIS seems mainly driven by its effectiveness, development and application of novel surgical techniques to treat patients with supratentorial spontaneous ICH with a potentially large effect on a clinical outcome is worthwhile also from a cost-effectiveness perspective.

## Conclusion

In patients with spontaneous supratentorial ICH, minimally invasive neurosurgery has the potential to be cost-effective, even with relatively low effectiveness. These results underline the need for a multicenter phase-III trial to investigate the effectiveness of MIS to improve an outcome after spontaneous supratentorial ICH. Based on these results, societal costs and patient burden for such a trial seem justified.

## Data Availability Statement

The original contributions presented in the study are included in the article/Supplementary Material, further inquiries can be directed to the corresponding author.

## Ethics Statement

Ethical review and approval was not required for the study on human participants in accordance with the local legislation and institutional requirements. Written informed consent for participation was not required for this study in accordance with the national legislation and the institutional requirements.

## Author Contributions

CK, FS, and JG concepted and designed the study. FS, JG, MU, and MS contributed to acquisition and analysis of data. CK, FS, JG, LS, and MS contributed to interpretation of data. FS, MU, and MS drafted the manuscript. CK, JG, LS, MR, and RD contributed to critical revision of the manuscript. CK and MR contributed to funding. CK, JG, and MR contributed to supervision of the study. All authors contributed to the article and approved the submitted version.

## Funding

This study was sponsored by the Netherlands Cardiovascular Research Initiative, which is supported by the Dutch Heart Foundation, CVON2015-01: CONTRAST and the support of the Brain Foundation Netherlands (HA2015.01.06). FS and CK are supported by the Dutch Heart Foundation (a clinically established investigator grant, 2012T077). CK is supported by ZonMw (ASPASIA grant, 015008048). FS is supported by the Dutch Heart Foundation (a senior clinical scientist grant, T2019T060). MS, MR, and JG are supported by a grant from the Dutch Research Council (No. 91818617).

## Conflict of Interest

The authors declare that the research was conducted in the absence of any commercial or financial relationships that could be construed as a potential conflict of interest.

## Publisher's Note

All claims expressed in this article are solely those of the authors and do not necessarily represent those of their affiliated organizations, or those of the publisher, the editors and the reviewers. Any product that may be evaluated in this article, or claim that may be made by its manufacturer, is not guaranteed or endorsed by the publisher.

## Footnotes

^1^https://www.exchangerates.org.uk/USD-EUR-spot-exchange-rates-history-2019.html#:~:text=Average%20exchange%20rate%20in%202019%3A%200.8931%20EUR (accessed on April 12, 2022).
