# Supplementary material for: Identifying the Conditions for Cost-Effective Minimally Invasive Neurosurgery in Spontaneous Supratentorial Intracerebral Hemorrhage
Source: Front Neurol. 2022 Jun 2;13:830614. doi: 10.3389/fneur.2022.830614 (PMC9200972; doi:10.3389/fneur.2022.830614)
Supplement: Supplementary file 1 [file Data_Sheet_1.PDF]

Supplementary Material

Supplemental Table 1: Unit cost prices

**Parameter**	**Unit price (in euro)**
Intensive Care Unit (per day)	2,152
Medium Care Unit (per day)	686
General Ward (per day)	422
Rehabilitation centre (per day)	491
Nursing home (per day)	219
Home care (per hour)	53
Ambulance (per ride)	290
Outpatients visits (per visit)	106
General Practitioner (per visit)	35
Total surgical costs for minimally-invasive surgery	10,000

Prices (in euro, price index 2019) per parameter. Data derived from [1]

Supplemental Table 2: Resource use in the first 3 months post-ICH, according to modified Rankin Scale score for 0-3 months post-ICH.

**Parameter**	**Data source**	**mRS 0**	**mRS 1**	**mRS 2**	**mRS 3**	**mRS 4**	**mRS 5**	**mRS 6**
LOS Intensive care unit (mean days, SD)	Radboudumc cohort	0.29 (0.83)	0.06 (0.24)	0.4 (1.3)	2.4 (6)	2.8 (7.2)	16 (21)	0.77 (3.1)
LOS Medium care unit (mean days, SD)	Radboudumc cohort	0.14 (0.36)	0.18 (0.53)	0.1 (0.45)	0.18 (0.4)	0.17 (0.71)	0.62 (1.4)	0.27 (1.5)
LOS general ward (mean days, SD)	Radboudumc cohort	5.2 (4.2)	4.5 (3.3)	7 (6.4)	12 (14)	11 (6)	15 (14)	3.5 (4.4)
LOS non-academic hospital (mean days, SD)	Radboudumc cohort	0	0	0.7 (3.1)	0	0.83 (2.6)	0	0.39 (2.1)
LOS rehabilitation center (mean days, SD)	Radboudumc cohort	24 (35)	13 (28)	19 (31)	26 (37)	32 (30)	35 (38)	2.1 (10)
LOS nursing home (mean days, SD)	Radboudumc cohort	0	4.9 (20)	4.8 (18)	18 (34)	14 (26)	22 (31)	3.4 (12)
Home care (mean hours, SD)	Christensen e.a. [2]	15.5 (66.7)	52.3 (118.6)	60.9 (136.3)	118.1 (211.3)	184.4 (184.4)	143.1 (280.7)	0.1 (1.9)
Ambulance (mean rides, SD)	Radboudumc cohort	1.4 (0.5)	1.3 (0.47)	1.6 (0.69)	1.6 (0.5)	2.1 (0.9)	2.2 (0.46)	1.2 (0.43)
Outpatient visits (mean amount visits, SD)	Radboudumc cohort	0.07 (0.27)	0	0	0.09 (0.3)	0	0	0
GP visits (mean amount visits, SD)	assumption	1	1	1	1	1	0	0
Medication use (mean in €, SE)	Spieler e.a. [3]	€162 (19)	€162 (19)	€162 (19)	€146 (27)	€129 (31)	€129 (31)	€0

Abbreviations: GP, general practitioner; ICH, intracerebral hemorrhage; LOS, length of stay; mRS, modified Rankin Scale; SD, standard deviation; SE, standard error.

Supplemental Table 3: Resource use between 3 and 12 months post-ICH (per 3 month cycle), according to modified Rankin Scale score.

**Parameter**	**Data source**	**mRS 0**	**mRS 1**	**mRS 2**	**mRS 3**	**mRS 4**	**mRS 5**	**mRS 6**
LOS rehabilitation center (mean days, SD)	Radboudumc cohort	1.1 (3.6)	0.02 (0.08)	0.62 (2.8)	5 (10)	6.3 (12)	12 (17)	0
LOS nursing home (mean days, SD)	Radboudumc cohort	0	2.9 (12)	11 (28)	16 (36)	20 (35)	66 (41)	0
Home care (mean hours, SD)	Christensen e.a. [2]	15.5 (66.7)	52.3 (118.6)	60.9 (136.3)	118.1 (211.3)	184.4 (184.4)	143.1 (280.7)	0.1 (1.9)
Outpatient visits (mean amount visits, SD)	Radboudumc cohort	0.12 (0.45)	0	0	0.12 (0.4)	0	0	0
GP visits (mean amount visits, SD)	assumption	1	1	1	1	1	0	0
Medication use (mean in €, SE)	Spieler e.a. [3]	€162 (19)	€162 (19)	€162 (19)	€146 (27)	€129 (31)	€129 (31)	€0

Abbreviations: GP, general practitioner; ICH, intracerebral haemorrhage; LOS, length of stay; mRS, modified Rankin Scale; SD, standard deviation; SE, standard error.

Supplemental Table 4: Resource use beyond 12 months post-ICH (per 3 month cycle), according to modified Rankin Scale score.

**Parameter**	**Data source**	**mRS 0**	**mRS 1**	**mRS 2**	**mRS 3**	**mRS 4**	**mRS 5**	**mRS 6**
LOS nursing home (mean days, SD)	Radboudumc cohort	0	0	15 (35)	0	6.2 (20)	60 (39)	0
GP visits (mean amount visits, SD)	assumption	1	1	1	1	1	0	0
Medication use (mean in €, SE)	Spieler e.a. [3]	€162 (19)	€162 (19)	€162 (19)	€146 (27)	€129 (31)	€129 (31)	€0

Abbreviations: GP, general practitioner; ICH, intracerebral haemorrhage; LOS, length of stay; mRS, modified Rankin Scale; SD, standard deviation; SE, standard error.

Supplementary references

1: Hakkaart-van Roijen L, Van der Linden N, Bouwmans C, Kanters T, Tan SS. Kostenhandleiding: Methodologie van kostenonderzoek en referentieprijzen voor economische evaluaties in de gezondheidszorg. [in Dutch]. 2015

2: Christensen MC, Morris S. Association between disability measures and short-term health care costs following intracerebral hemorrhage. Neurocrit care. 2008;9(3):313-8.

3: Spieler JF, Lanoe JL, Amarenco P. Costs of stroke care according to handicap levels and stroke subtypes. Cerebrovasc Dis. 2004;17(2-3):134-42.
